# Seroconversion rates following 2 doses of measles- mumps- rubella vaccination given at the ages 12 and 18 months: data for possible additional dose at older age

**DOI:** 10.1186/s12865-021-00465-1

**Published:** 2022-01-16

**Authors:** Hana Saffar, Sayed Jaber Mousavi, Hiva Saffar, Mohammad-Reza Parsaei, Gholam-Reza Ghorbani, Mohammad Jafar Saffar

**Affiliations:** 1 Department of Anatomical and Clinical Pathology, IKHC, Teheran University of Medical Sciences, Tehran, Iran; 2 Department of Community Medicine, Faculty of Medicine, Mazandaran University of Medical Sciences, Sari, Iran; 3 Department of Pathology, Shariati Hospital, Teheran University of Medical Sciences, Tehran, Iran; 4 Deputy of Health, Mazandaran University of Medical Sciences, Sari, Iran; 5 Pediatric Infectious Diseases Research Center, Communicable Diseases Institute and Department of Pediatric Infectious Diseases, Bu-Ali Sina Hospital, Mazandaran University of Medical Sciences, Pasdaran Bolv, Sari, Iran

**Keywords:** Seroconversion rate, MMR, Iran, Measles, Mumps, Rubella, Elimination, Primary vaccine failure

## Abstract

**Background:**

Despite high rate of vaccination coverage with 2-doses of measles containing vaccine among Iranian children, outbreaks of measles occurred among different age groups and fully vaccinated subjects. Although the main reason for these outbreaks is unknown, however, vaccine failure was supposed to be an important cause. This study was designed to determine the seroconversion rates to measles- mumps- rubella (MMR) vaccine currently in use among Iranian children.

**Methods:**

This prospective study was conducted among healthy children older than 12 months who were candidates of scheduled MMR vaccination. Blood samples were obtained from each mother- infant pair just before vaccination, and from infants 4–6 weeks after MMR_1_ and MMR_2_ immunization. Collected sera were tested for specific lgG antibodies against MMR agents using ELISA method. The proportion of seroprotected subjects among mother- infant pairs before vaccination as well as the prevalence rates of seroconversion after MMR_1_ and MMR_2_ vaccination were calculated. Collected data were analyzed using descriptive statistical methods.

**Results:**

During 22-months study period, 92 mother- infant pairs were participated. Seroimmunity rates against MMR viruses were 85.8%, 84.7% and 86.9% for mothers, and 3.2%, 2.1% and 1.0% for children, respectively. After MMR_1_ vaccination from 52 seronegative children, 80.7%, 78.8% and 75% were seroconverted. These rates increased to 94.8%, 89.7% and 94.8% after the MMR_2_ vaccination. Also, the specific immunity was enhanced among seropositive children.

**Conclusion:**

Majority of the mothers and few infants were immune to MMR viruses prior to MMR_1_ vaccination. Immune responses detected after MMR_1_ injection, and overall seroconversion rates achieved after 2-doses of MMR vaccination were less than expected and inadequate to preserve long-term protection against MMR agents.

## Background

Measles, mumps, and rubella (MMR) are communicable viral illnesses that are preventable through vaccination. Measles is a highly contagious infection that can be transmitted to more than 90% of susceptible subjects and is still a major cause of death among children, particularly in children less than 5 years old [1]. About 140,000 measles deaths were reported worldwide in 2018 [2]. From first of January-to the end of July of 2019, more than 364,806 cases of measles were reported from 182 countries in the world; this rate surpassed 23% from the similar period in 2018 [2, 3]. Mumps, an acute contagious disease, most often affects susceptible children to young adults in the closed crowded community and can be associated with serious complications such as: meninigoencephalitis, orchitis, pancreatitis, myocarditis, and nephritis [4, 5]. Rubella, a mild exanthematous illness, can be a real threat when infecting pregnant women especially in the first trimester and can result in fetal loss or devastating multiple anomalies known as congenital rubella syndrome (CRS) [6, 7].

According to the World Health Organization (WHO) and other authorities, all children should receive a second dose of MMR vaccine with the aim of reduction in proportion of primary vaccine failure (PVF) in susceptible individuals and also this act as an essential strategic key measure of measles elimination [1, 8–11].

Following universal two doses scheduled monovalent measles vaccine (mMV) immunization of the Iranian children from 1984, and the national measles—rubella (MR) campaign of the 5- 25 year- old population in December 2003, the incidence of measles cases in the Iran reduced markedly [12, 13]. However, despite the high vaccine coverage rates, during recent years small outbreaks of measles have occurred even among fully vaccinated subjects in some regions of the country. The true reasons for these outbreaks are not clear. However, for more description, a brief epidemiological characteristics of reported measles outbreaks from the year 2006 are presented in Table 1 [14–18].

**Table 1:** Epidemiological characteristics of measles outbreaks reported in Iran from years 2006 to 2016

Author/ reported province	Years of outbreaks	No of cases	Involved age groups	Vaccination status
Nejati et al. [14] Sisdtan- Bluchestan	2006–11	456 cases	All age groups: 48% in < 5 yrs 34% in 5–9 yrs	One dose: 11.2% 2-dose: 27.8%, not vaccinated: 55.1%
Izadi et al. [15] southeast of Iran	2009–10	126 cases (2 main outbreaks)	All age groups 42% ≥ 7 yrs 6.3% > 20 yrs	2-doses vaccine efficacy:74.2%
Moghaddam et al. [16] Fars	2012	7 cases	11 months–35 yr	2-cases: unknown 3cases full vaccinated 2 cases Non-vaccinated
Karami et al. [17] National level	2012 and 2014	2012:232 cases 2014: 142 cases	All age groups	22.7%: < 12 mo 19.3%: vaccinated 36.5%: non-vaccinated
Piri et al. [18], National level	2014–2016	759 cases	31.1%: < 1 yr 13.2%: 1–4 y 11.6%:5–9 y	< 1 yr: 0.9% vaccinated 1–4 yr: 9% vaccinated 5–9 yr: 7.1% vaccinated 23.3%: vaccinated

2–14 years after the national MR immunization program of 5–25 years-old population and the onset of two-dose MMR immunization program of ≥ 12 months old children in the country

Previous measles seroprevalence studies conducted in the Iran showed a gap between immunization coverage rates and the proportion of those who were seropositive [19]. The extent of this gap is influenced by several factors including age at the time of initial immunization and time elapsed since the last vaccination [20–22], as well as vaccine related factors such as: type of the virus strain and cold-chain regulation in health care centers [23]. In order to determine the immunogenicity of MMR vaccine among Iranian children, some studies were performed. The seroconversion rates against MMR agents observed in these studies varied markedly [24–27]. While in one study from Ahwaz, 6 months following scheduled MMR vaccination of older than 12 month-old children, the detected seroconversion rates were 42.9%, 58.6% and 90%, against MMR agents [24], in another similar study from Tehran, these rates were 75.8%, 95.3% and 78.3%, respectively [25]. These findings raised concern about possible role of PVF as a cause of measles outbreaks occurred in fully vaccinated individuals, possibly because of poor control of cold-chain and vaccination procedures, stunting and/or possible persistence of specific antibody beyond 12 months of age [15, 16, 24, 26, 27].

This study was designed to investigate the seroconversion rates of the MMR vaccine currently in use among a cohort of more than 12 month-old children following 2 doses of MMR vaccine given at the ages of 12 and 18 months. Also, the seroprevalence rates of specific immunity to MMR agents among children just before their immunization and its possible effects on the vaccine immunogenicity were determined.

## Methods and subjects

Over a 22-month study period (1 October 2017 to 31 July 2019), healthy children aged ≥ 12 month-old who were brought to the primary health care centers for scheduled MMR immunization program in East of Mazandran province, North of Iran were recruited on a voluntary basis. Children with acute illness, those with history of chronic diseases, or malignancies/ immunodeficiency, history of febrile exanthematous diseases or recipients of blood/ blood products/additional MMR or any measles containing vaccines, those with history of prematurity (gestational age < 36 weeks and/or birth weight < 2500 g), and any contraindications to MMR vaccination were excluded. Standard ethical guidelines and protocols were used and the study was approved by the Ethic Committee of the Mazandran University of Medical Sciences; IR. MAZUM.REC1390.3073. After obtaining informed written consent from guardian, 3 ml (ml) blood was obtained from each infant- mother pair. Four- to 6 weeks after vaccination with MMR vaccine (MMR_1_): [Domestic Razi institute of serum and vaccines: Karaj- Iran (Measles: AIK- C, Mumps: Hoshino, Rubella: Takahashi all CCID50 1000), or MMR vaccine: Serum Institute of India: (Measles: Edmonston- Zagreb; CCID50 1000, Mumps; Leningrad- Zagreb CCID50 5000; Rubella wistar RA27/5 CCID50: 1000)], 3 ml of venous blood was collected from vaccinated infants. Vaccines were dispensed into multidose (2, 5 dose/vial) and were stored at 2–8 °C and reconstituted before vaccination. Reconstituted unused vaccines were discarded after 6 h. Collected sera were stored at − 20 °C. Based on the objectives of the study, no intervention was done during vaccination procedures by researchers. According to the National Immunization program in Iran, these children came back for scheduled second dose of MMR vaccine (MMR_2_) after 6 months. 4 to 6 weeks after MMR_2_, third venous blood samples were obtained. All collected sera (mother and infant pairs) were tested for specific immunoglobulin G (lgG) against measles, mumps and rubella with ELISA methods, using semi quantitative Vircell microbiologist (measles lgG/lgM, G/M 1001, mumps lgG/lgM, G/M 1014, and Rubella lgG, G/ 1026) ELISA Kits (Vircell, S.L. Parquet Technologico dela salud. Avecina 8.10.016 Granada, Spain), in the University laboratory.

Based on the manufacturer`s instructions the results were interpreted as antibody index. The antibody indices were calculated with positive and negative controls (OD > 0.9 and OD < 0.5) at a control cut-off range of > 0.55 to < 1.5. Antibody indices were measured as: (sample OD/cut-off serum mean OD) × 10. Samples with antibody indices < 9 were considered as negative (not containing protective titers of specific antibody), 9–11 equivocal and > 11 as positive (seroprotected and immune). Equivocal samples were rechecked, and if < 11, called as negative and if > 11, interpreted as positive. This categorization was applied for three MMR agents. The rubella ELISA has been standardized against WHO first international standard for anti-Rubella IgG with a cut-off set at 10 IU/ml. The seroconversion rates against each agent among mother-infant pairs were calculated before vaccination. After MMR_1,_ the proportion of responders and the mean concentration of antibodies (MCAs) for each agent were determined among seroconverted children. Also in order to determine the possible influence of maternal antibodies, immunologic responses to MMR_1_ vaccination and the proportion of responders and the acquired MCA levels, were compared between two groups of children including those with seropositive mothers and those without such history. Then, the proportion of seroconveted subjects among nonresponders to MMR_1_ and the MCA levels of both new responders and seroimmune subjects were calculated following MMR_2_ vaccination. Collected data were analyzed using SPSS version of 16.0. The descriptive statistical method was used in the form of percentile for seroconversion and response rate, and Chi-Square and students T-test were applied to find differences between variables as appropriate. Results were considered to be statistically significant when the P value was less than 0.05.

## Results

For this study, during 22 months of study period, 92 mother- infant pairs were participated. Of 92 infants, 43 were females and 49 males; the mean age of mothers was 27.4 years (age range 21–42 years) and the mean age of children for MMR_1_ and MMR_2_ vaccination was 12.1 and 18.3 months, respectively. After the MMR_1_ vaccination, out of 92 participated children, 52 (56.5%) were willing to continue the study and blood samples were taken from them. Also, after MMR_2_ vaccination, 39 (42.4%) participants agreed to give blood samples. As are presented in Table 2, approximately, 85.8%, 84.7% and 86.9% of mothers serologically were immune against MMR agents. These rates were 3.2%, 2.1%, and 1.0%, respectively for children just before the first dose of MMR injection.

**Table 2:** Anti-Measles- Mumps- Rubella seroprevalence profiles among studied mother-infant pairs just before MMR immunization, seroconversion rates and mean concentration of antibody levels following first and second dose of MMR vaccine given at the ages 12 and 18 months, Sari-Iran 2018

MMR vaccination status	Measles: n (%)	MCA ± SD	Mumps: n (%)	MCA ± SD	Rubella: n (%)	MCA ± SD
Pre-MMR_1_ vaccination						
Mothers n = 92	79 (85.8%)	22.40 ± 7.25	78 (84.7%)	21.30 ± 5.76	80 (86.9%)	19.18 ± 4.32
Infants n = 92	3 (3.2%)	–	2 (2.1%)	–	1 (1.0%)	–
Post MMR_1_ n = 52	44 (84.6%)	22.20 ± 6.35	43 (82.7%)	18.40 ± 5.15	41 (78.8%)	21.30 ± 5.76
Post MMR_2_ n = 39	37 (94.8%)	28.44 ± 6.17	35 (89.7%)	26.67 ± 5.80	37 (94.8%)	27.08 ± 7.68
No of responders /no of susceptible (%)	4/6 (66.6%)		3/7 (42.8%)		8/10 (80%)	
Comparison between post MMR_1_ and MMR_2_ MCA levels; P value	P = 0.003		P < 0.001		P < 0.001	

After administration of MMR_1_ vaccine, nearly 84.6%, 82.7% and 78.8% of seronegative vaccinated children responded to MMR agents of vaccine and became IgG seroconverted. None of the infants who were seropositive before MMR_1_ vaccination, were included in the further stages of study. The MCAs levels for three MMR agents were 22.20 ± 6.35, 18.40 ± 5.15 and 21.30 ± 5.76, respectively. When these rates were compared between two-groups of children (those born from seropositive mothers and those without this history), despite small sample size and lack of adequate power, no statistically significant increased rate of seroconversion or MCA levels was identified in children of seronegative mothers (Table 3). After the MMR_2_ injection, of 39 studied children, 6 were susceptible to measles and 4 subjects responded to MMR_2_ and the total number of seroconverted children against measles reached to 37/39 (94.8%). These rates were 7 and 3 (35/39;89.7%), 10 and 8 (37/39; 94.8%) for mumps and rubella respectively. The acquired MCAs levels were significantly higher than those after MMR_1_, indicating enhancement of immunity. MCA was, 28.44 ± 6.17 VS 22.20 ± 6.35 P = 0.003 for measles, 26.67 ± 5.80 VS 18.40 ± 5.15, P < 0.001for mumps, and 27.08 ± 7.68 VS 21.30 ± 5.76, P =  < 0.001 for rubella. All of these collected data are presented in Table 2.

**Table 3:** The patterns of infants’ immune response to MMR_1_ immunization in relation to their mother specific antibody status

	Infant mother antibodies status prior to MMR_1_ injection		
	Antibody positive	Antibody negative	P value
Measles; N = 52	43	9	
Response rate (%)	83.72%	88.88%	0.6
MCA level	21.08 ± 5.75	23.25 ± 5.03	0.34
Mumps; n = 52	43	9	
Response rate (%)	81.81%	87.50%	0.8
MCA level	17.78 ± 4.31	18.66 ± 5.21	0.58
Rubella; n = 52	45	7	
Response rate (%)	77.77%	85.7%	0.63
MCA level	20.23 ± 6.37	22.33 ± 4.72	0.44

## Discussion

In this study, nearly 85% of investigated mothers were serologically immune to MMR viruses. Few more than 12 month-old infants also showed seroprotection against these agents before their scheduled MMR_1_ vaccination. After administering the MMR_1_ to more than 12 month-old children, nearly 84, 6%, 82.7% and 78.8% of vaccinated children responded to three components of MMR_1_ vaccine, and were seroconverted, respectively. However, these rates, were higher in infants of seronegative mothers. Six months after the initial MMR immunization, most serosusceptible children showed immunologic response to MMR_2_ and serologically achieved protection to MMR agents. Also, their earlier acquired seroimmunity following the first dose of MMR vaccine injection was enhanced. Finally, following administering 2- doses of MMR vaccine to children after the age of 12 months, approximately 94.8%, 89.7% and 94.8% of vaccinees acquired seroprotection to measles, mumps, and rubella, respectively.

Based on this study findings, most participated mothers were seropositive against three agents of vaccine. In addition to history of measles immunization during childhood, majority of our studied mothers were reimmunized in the national program of MR campaign conducted at the December 2003 [12]. However, none of these mothers were immunized against mumps earlier and their seroimmunity against mumps is the results of natural infection. The high rates of seropositivity against measles and rubella may be due to positive impact of MR reimmunization performed 13 to15 years earlier or it could be the result of exposure to natural infection in the past. Similar results are reported among Iranian childbearing age women with the same age recently. In a nationwide seroprevalence study among Iranian girls at the verge of marriage whom were MR reimmunized 13–14 years earlier, results showed that 80.7% (70.3-to 89.5%) and 90.6% (81.2-to 95%) were immune to measles and rubella, respectively. There was a sharp difference between those who were MR vaccinated and those who were not. They concluded that these high rates of seroimmunity were the positive impact of MR revaccination [28]. However, these antibodies disappear during first few months of life, higher levels will persists for a longer time [29].

According to our data, using ELISA method for antibody measurement, a few children were seroimmune to MMR agents just before MMR_1_ vaccination. The exact origin of these antibodies is not clear, but most probably is maternal. In a similar study from the region among 112 mother-infant pairs in year 2008 (5-years after the national MR campaign), nearly 6.2% and 10.7% of 12 month-old infants, all from MR reimmunized mothers, retained their seroimmunity to measles and rubella, respectively [27]. Also, in another similar study from southeast of Iran, using ELISA method, nearly 3.7% of 12 month-old infants were serologically immune to measles just before MMR_1_ vaccination [26]. The point of concern is that ELISA method is not sensitive enough to detect low titers of measles antibodies [30]. Therefore, if a more sensitive method has been used, probably a higher proportion of infants would have shown specific immunity (retained their passive immunity) just before MMR_1_ immunization. Since MMR vaccine is a live attenuated vaccine, the presence of specific antibody may have a negative influence on the immunogenicity of the vaccine. This assumption was confirmed by studies conducted in the 1990s [31–33]. In a serological study, using a sensitive virus neutralization test, it was found that many 12 month-old children had preexisting maternal antibody to measles virus and their immunological response to measles vaccination was affected [31]. In this study, after giving scheduled MMR1 vaccine to more than 12 month-old seronegative children, nearly 15%, 17%, and 21%, of vaccinated children remained seronegative to MMR agents (PVF), respectively. When these results were compared between 2 groups of children, these rates were higher in children of seropositive mothers.

Other researchers have also investigated the immunogenicity of MMR vaccine among Iranian children older than 12 months [24–27]. Most of these studied results showed a lower than expected seroconversion rates, even after strict control of vaccination procedures and cold-chain regulation. The rates of PVF detected in Iranian studies were higher than the rates reported worldwide. The main possible reason for the higher rates of PVF observed in our study as well as other Iranian studies may be due to presence of low concentrations of specific antibodies, particularly anti-measles antibody, undetectable by ELISA method, and its negative influence on the immunogenicity of the MMR vaccine [31–33]. However, the possible negative impact of technical problems in vaccination procedure should be also considered [15, 16, 24–27]. Further studies are recommended to investigate the presence of specific antibodies, particularly anti-measles antibody by a more sensitive method just before MMR_1_ immunization at the age 12–13 months as well as its effects on the immunogenicity of MMR vaccine. Furthermore, periodic educational sessions are suggested for vaccinators to improve vaccination techniques, particularly cold-chain regulation.

After administering the MMR_2_ vaccine to 18 months old previously MMR_1_ vaccinated children, most susceptible ones were acceptably seroconverted. Also, their earlier acquired immunity was enhanced. However, the overall seroprotection rates detected in this study following 2-doses of MMR vaccine were lower than expected in the world. Results of most studies in the world indicates that vaccination with 2-doses of MMR vaccine after the age of 12 months is associated with > 95–98% seroconversion rate [1, 8–11, 20–22], but the results of most studies reported from Iran are varied and show lower rates than the global results [19, 24, 34]. While the result of one study from north of Iran showed seroconversion rates of 98.2% and 94.4% for measles, 92.4% and 87% for rubella component after MMR_2_ vaccine given at the age of 6 years or 18 months [34], in another study from Ahwaz 6 months after MMR vaccination of 6.5 year-old children with history of 2-doses of Mmv at the ages of 9 and15 months, the seropositivity rate was 45.6%, for measles,76.7% for mumps and 87.7% for rubella [24]. However, our data in this study indicated acceptable, but not optimal seroconversion rates among Iranian children following two-doses of MMR immunization.

Considering these rates of seroprotection along with 97% vaccination coverage rate in all districts of the country, a population immunity rate of about 91.9%, 87.0% and 91.9% for MMR agents could be estimated, respectively. The concerning point is that vaccine-induced antibodies against measles and mumps decrease faster over time comparing to rubella [35–37]. Therefore, an increasing numbers of potentially measles-mumps susceptible population will accumulate in the community and facilitate outbreaks even among fully vaccinated subjects [38–40]. However, this rate of immunity against measles is lower than that is required in a community to eliminate/sustain measles elimination [1, 8–11]. The rates for mumps and rubella are at the lowest threshold to eliminate mumps and rubella epidemics [4, 8]. To prevent the measles virus transmission in a community, a population immunity rate of 93% to 95% with > 95% two-dose vaccine coverage rate is required in all districts of the country. This level for rubella and mumps is estimated as 88% to 90% [8–11]. Therefore, these levels of immunity are challenging in mid- to long-term period and raise concern about the sustaining measles-rubella elimination in the country which has received certificate of elimination in the last 2 years [41]. While considering our data and other Iranian studies reports, periodic serosurveillance are recommended to monitor population immunity against MMR. Further studies with larger sample size, using more sensitive laboratory methods to measure low levels of specific antibodies just before MMR_1_ immunization along with strict control of cold-chain regulation and vaccination procedures in primary health care and vaccination centers are suggested. If these results are confirmed by further studies, changing the age of the first dose of MMR vaccine to 14 to15 months and/or considering additional universal dose of MMR vaccine at the older age are recommended.

The main limitations of this study include its small sample size and also using two brands of MMR vaccine interchangeably that may influence the final results.

## Conclusion

Based on the study findings, the seroconversion rates detected following two-doses of the MMR vaccine currently in use in the country is acceptable in short-term. However, to maintain mid- to long-term herd immunity, national or regional supplementary immunization activities seem reasonable. Similar studies with larger sample size in different regions of the country are recommended to measure specific antibody concentration particularly measles antibody with a more sensitive method before first dose of MMR administration, and also consider strict control of cold-chain and vaccine administering techniques to assess the MMR vaccine immunogenicity.

## Data Availability

The datasets used and/or analyzed during the current study are available from the corresponding author on reasonable request.

## Abbreviations

MMR: Measles mumps rubella
ELISA: Enzyme-linked immunosorbent assay
CRS: Congenital rubella syndrome
WHO: World health organization
PVF: Primary vaccine failure
SVF: Secondary vaccine failure
mMV: Monovalent measles vaccine
MAZUMS: Mazandaran University of Medical Sciences
IgG: Immunoglobulin G
MCA: Mean concentration antibodies
MR: Measles-rubella
ml: Milliliter
OD: Optical density
